# The Major Depression Inventory for diagnosing according to DSM‐5 and ICD‐11: Psychometric properties and validity in a Swedish general population

**DOI:** 10.1002/mpr.1966

**Published:** 2023-04-12

**Authors:** Andreas Lundin, Jette Möller, Yvonne Forsell

**Affiliations:** ^1^ Department of Global Public Health Karolinska Institutet Stockholm Sweden

**Keywords:** diagnostic test accuracy, DSM‐IV, ICD‐10, MDI, sensitivity and specificity

## Abstract

**Objectives:**

The Major Depression Inventory (MDI) was constructed to assess DSM‐IV and ICD‐10 depression symptoms, and does not fully cover the symptoms listed in DSM‐5 and ICD‐11. This study aimed to augment the MDI to the new diagnostic standards by adding a new item, and to assess and compare the measurement performance of the MDI items and diagnostic algorithms for major depression according to DSM‐IV, ICD‐10, DSM‐5 and ICD‐11.

**Methods:**

Surveys collected 2001–2003 and 2021, including self‐assessed MDI were used. A new hopelessness item was constructed and analyzed alongside the hopelessness item in the Symptom Checklist. The performance of items was compared using Rasch and Mokken analyses. Criterion validity was examined using equivalent diagnoses from psychiatric interview (Schedules for Clinical Assessments in Neuropsychiatry [SCAN]) as standard.

**Results:**

MDI information was provided by 8511 individuals in 2001–2003 (SCAN subsample *n* = 878), and 8863 in 2021. All items, including hopelessness had good psychometric properties. Sensitivity ranged between 56% and 70%, and specificity between 95% and 96%, indicating similar criterion validity.

**Conclusions:**

Hopelessness and the MDI items had good psychometrics. MDI for DSM‐5 and ICD‐11 had similar validity as for DSM‐IV and ICD‐10. We recommend that MDI is updated to DSM‐5 and ICD‐11 by adding a hopelessness item.

## 1 INTRODUCTION

The Major Depression Inventory (MDI) was constructed by Per Bech and colleagues to cover symptoms assessing DSM‐IV Major Depression and ICD‐10 Major Depressive Disorder (Bech et al., 2001) and is used both clinically (Danish Health Authority, 2007; Nielsen et al., 2017) and in surveys (Andersen et al., 2011; Christensen et al., 2017; Ellervik et al., 2014; Forsell, 2007; Otiende et al., 2017a). The inventory contains 13 questions which can be dichotomized to correspond to the ten ICD‐10 and nine DSM‐IV symptom criteria needed to assess DSM‐IV and ICD‐10 operationalization of depression. The individual MDI items has shown to have good reliability (Olsen et al., 2003; Otiende et al., 2017b), and the algorithmic classifications acceptable sensitivity and specificity for detecting DSM‐IV and ICD‐10 depression in the general population (Forsell, 2005), primary care (Nielsen, et al., 2017) and psychiatric care (Bech et al., 2001).

In 2013 the fifth revised DSM version (DSM‐5) was released, and in 2020 the first part of the eleventh revised version of ICD (ICD‐11), both of which contained changes in the operationalization of Major Depression. DSM‐5 remained close to DSM‐IV; the criteria are still nine and a diagnosis require at least five of these to be present, of which one must be low mood or loss of interest. However, a seemingly small change in wording expanded the core mood symptom to also include hopelessness (the subjective description of the sadness symptom now reads “e.g. feels sad, empty, hopeless”) which potentially broadens this symptom and in the end the diagnosis (Uher et al., 2014). ICD‐11 criteria (here called features), similar to those of the ICD‐10 Clinical descriptions and diagnostic guidelines (CDDG) or ICD 10 Research Diagnostic Guidelines (RDG) has not yet been released, but the upcoming changes have been presented in other forms (Stein et al., 2020) and are extensive: Hopelessness appear as a new (accompanying) feature; low energy is changed from a core to an accompanying feature, and; worthlessness and guilt are combined into a composite feature. While the number of symptoms remains ten, the threshold is raised from at least four to at least five to be diagnosed with depression, of which one needed to be a core symptom and not 2 as in ICD‐10. With the exception of the new criteria hopelessness, the changes make ICD‐11 more consistent with DSM.

While hopelessness is new in the DSM and ICD nosological systems it is not a new depressive symptom. It is commonly included in depression scales, as an aspect of mood/affect (e.g. Hamilton Depression Rating Scale (Hamilton, 1967), PHQ‐9 (Kroenke et al., 2001)), or a separate cognitive symptom (e.g. CES‐D (Radloff, 1977), Zung Depression Scale (Zung, 1965), and Beck Depression Inventory‐II (Beck et al., 1961)). Hopelessness also appeared already in the ICD‐10 CDDG (but not in the RDG (WHO, 1993)) listed as an accompanying symptom, and described as “bleak and pessimistic views of the future” (p, 119) (WHO, 1992). In studies used to justify the inclusion of hopelessness in ICD‐11, at the time current DSM‐IV criteria were compared to non‐current symptoms, showing that hopelessness had better psychometric properties than many of the current criteria (McGlinchey et al., 2006; Stein, et al., 2020). In psychological, cognitive theories on depression, hopelessness is a central feature, for example, the hopelessness theory of depression and its' specific subtype hopelessness depression, and Beck's cognitive theory where hopelessness comprises “a major feature of the symptomatology of depression” (Beck et al., 1988).

The lack of a hopelessness item in MDI hampers assessment in relation to ICD‐11 or DSM‐5 depression. To address this deficit we formulated such an item and embedded it in relation to other MDI items in a new (2021) wave of the population‐based cohort included in the PART study (Swedish acronym for Mental Health, Work and Relations). The MDI is used throughout all waves of PART, but in one wave (2003) it was included in parallel with depression symptoms from the Symptom Checklist, SCL (Derogatis et al., 1974) which include an item on hopelessness. Setting out from this hopelessness item we modified the MDI diagnostic algorithms to encompass DSM‐5 and ICD‐11 operational criteria. A subset of the 2001–2003 wave also participated in a psychiatric interview covering DSM‐IV and ICD‐10 symptoms and non‐current symptoms, including hopelessness. This information was used to compare the agreement between MDI depression according to algorithms for DSM‐IV, DSM‐5, ICD‐10 and ICD‐11 to their equivalent clinical diagnoses. The cohort baseline‐data has previously been used to validate the MDI algorithm for DSM‐IV depression as well as the MDI rating scale (Forsell, 2005).

The current study set out to first compare the relative performance of hopelessness, as assessed through the newly formulated item and through proxy of the SCL item. Secondly, the criterion validity of the existing MDI algorithms (DSM‐IV, ICD‐10) was assessed together with new algorithms (DSM‐5 and ICD‐11).

The specific research questions are:

‐ What are the psychometric properties of hopelessness in relation to current MDI items?
‐ What are the agreements across MDI‐DSM‐IV, ‐DSM‐5, ‐ICD‐10 and ‐ICD‐11 depression?
‐ How does MDI‐DSM‐IV, ‐DSM‐5, ‐ICD‐10 and ‐ICD‐11 depression agree with equivalent past year diagnosis (criterion validity)?

## 2 METHODS

### 2.1 Population

**FIGURE 1:**
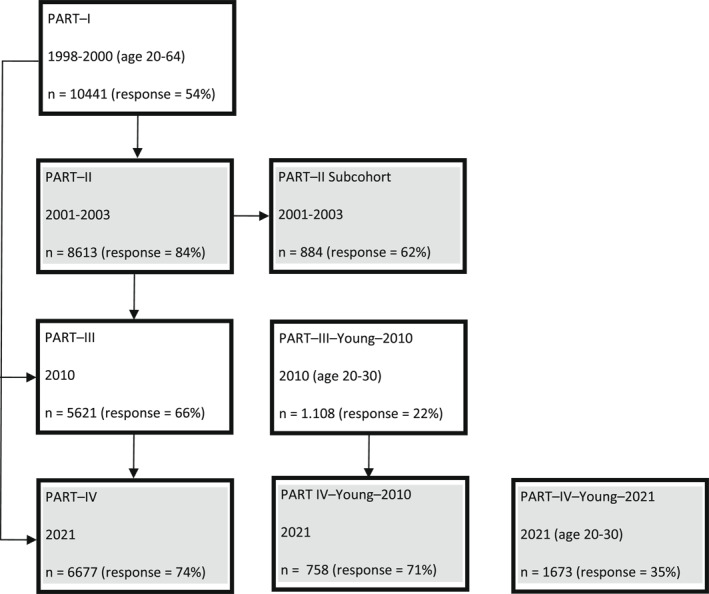
Flowchart of the PART data collection. In gray is indicated which data sets is used.

We used the first and fourth follow‐up waves of the Mental Health, Work and Social Relations study (PART by Swedish acronym). PART was first set up between 1998 and 2000 as a simple random sample of 20–64 year old in Stockholm County, whom were sent a postal questionnaire. Follow up questionnaires were sent out in 2001–2003 (PART‐II), in 2010 (PART‐III) and in 2021 (Part‐IV). A flow chart showing respondents and response rates are shown in Figure 1. As part of the 2010 and 2021 collections new samples of young adults (20–30 year old) included. In 2021 a first follow up was conducted on the 2010 young adults. A sub‐cohort (double‐phase stratified random sample) in PART‐II was invited to a psychiatric interview (*n* = 881), where those with low self‐reports on WHO‐10 wellbeing index (Bech et al., 1996) in the postal questionnaire were sampled at a higher probability (Lundin et al., 2015). The 2001–2003 sample contained 4915 women (58%) and 3596 men (42%). Means age was 45.1 years (SD = 12.4, range 22–68). The 2021 sample contained 5175 women (58%) and 3688 men (42%) with full MDI information. Mean age was 54.9 years (SD = 18.41, range 20–88). Of these 59% had a higher education (basic, advanced or doctoral), 33% an upper‐secondary education (2–4 years, vocational or preparatory higher education) and 8% comprehensive school (9 years compulsory school). Labor force status, reported in partially overlapping categories, was: employed (60%), retired (35%), student (8%), unemployed (4%), leave of absence (2%), sick‐leave >1 month (2%), and disability pension (2%).

### 2.2 Measures

#### 2.2.1 The Major Depression Inventory

The Major Depression Inventory (MDI) was included in the postal questionnaire and covered symptoms in the past 2 weeks. Each question asks about the amount of time that a symptom has been present, with six response alternatives: “All of the time”, “Most of the time”, “Slightly more than half of the time”, “Slightly less than half of the time”, “Some of the time” and, “None of the time”.

For the diagnostic algorithms the first three symptoms (mood, diminished interest and low energy) are dichotomized as at least present All or Most of the time, while the other symptoms are dichotomized as at least present Slightly more than half of the time (Bech, et al., 2001).

ICD‐10 depression diagnosis requires 4 out of 10 symptoms of which at least two are mood, diminished interest or low energy. DSM‐IV requires that 5 out of the 9 (low confidence and guilt are considered as one symptom), symptoms should be present, of which one is mood or diminished interest. For ICD‐11 hopelessness was added to the list of accompanying symptoms (dichotomized as for the accompanying symptom) and low confidence and guilt are considered as one symptom similar to DSM‐IV. Low energy was changed to an accompanying symptom (but still dichotomized as a core symptom, following the logic of the manual for DSM‐IV). The threshold was raised to five out of 10 symptoms, of which only one should be mood or diminished interest. The MDI DSM‐5 algorithm used was similar to that for DSM‐IV, but the score for mood and hopelessness (dichotomized as a core symptom) was used.

#### 2.2.2 Hopelessness

To include hopelessness we used the Symptom Checklist (Deragotis et al., 1973) which was used in parallel to MDI. The SCL asks about bothering symptoms in the past week and include a question on “Feeling hopeless about the future” for which the response alternatives were: “Not at all”, “A little bit”, “Moderately”, “Quite a bit”, and “Extremely”. The SCL depression subscale has previously been found to correlate strongly with the MDI rating scale (*r* = 0.79) (Cuijpers et al., 2007). In order to follow the MDI scoring logic we examined the response alternatives endorsement of other SCL items that have a similar content and wording as MDI items, concluding that MDI responses “All of the time” and “Most of the time” could be equated with SCL “Extremely” and “Quite a bit” for treating hopelessness as a core symptom in DSM‐5. For ICD‐11, where hopelessness is an accompanying symptom dichotomization was decided as “Quite a bit”, and “Extremely”, in order not to be too inclusive—this was done by examining the item endorsement of four other items that overlapped in MDI and SCL (sadness, anhedonia, low energy and feeling worthless), but also based on Rasch analysis.

A new question on hopelessness was constructed for the 2021 questionnaire. The Schedules for Clinical Assessments in Neuropsychiatry (SCAN) Glossary section provided a conceptual definition: “the person's view of the future is bleak and without comfort (…)”, which is consistent with ICD‐10 CDDG (WHO, 1992) and the ICD‐10 Glossary (Isaac et al., 1994). We interpreted it as consistent with the third concept in Beck's cognitive triad, that is, negative views about the future as distinct from negative views of the world or and from negative views on self (worthless). We reviewed several scales of depression, distress and psychological well‐being and hopelessness for items that contained words of “hopelessness”, “hopeless”, “hope”, “hopeful” and “future” to deductively create an item pool. The form, wording and responses were made to be consistent with the MDI format: negative worded, questions rather than statements and the six duration‐type response categories. A consensus was reached in a group comprising a psychologist, a psychiatrist, a public mental health epidemiologist with the formulation: “Have you felt that life is hopeless, your future appears bleak?” (in Swedish “Har du känt att livet är hopplöst, din framtid ter sig mörk?”). The question specifies that that it’s the individual's negative feelings about the own future, refers to a broad situation (life, no specific situation) and contain the key and commonly understandable concept of hopelessness.

#### 2.2.3 Psychiatric interview as index criterion

The SCAN version 2.1 (Wing et al., 1990), a semi‐structured interview intended for use by physicians, psychologists or mental health nurses, who have undertaken training at a World Health Organization designated SCAN training center, was used to assess clinically relevant symptoms. SCAN was originally developed to assess older system symptoms and criteria, for example, Feighner Criteria, Research Diagnostic Criteria (RDC) and DSM‐III and symptoms were added to encompass subsequent systems (e.g., ICD‐10 and DSM‐IV) but older symptoms were retained. Importantly, hopelessness is rated as a separate symptom. Based on the diagnostic algorithms we assessed depression according to DSM‐IV (APA, 1994), DSM‐5 (APA, 2013), ICD‐10 and the ICD‐11 as proposed by Mario Maj (Chairman of the Working Group on Mood and Anxiety Disorders for the ICD‐11) in Stein et al. (Stein, et al., 2020). The diagnoses did not consider bereavement or functional impairment because those are dissimilar between the diagnostic symptoms, and we were interested in the symptom‐sets. The items used in SCAN are provided in Supplementary Table (S1) for replication.

### 2.3 Statistical analyses

The performance of the MDI items was assessed using Rasch and Mokken analyses. Items were dichotomized according to the ICD‐10 dichotomization of core and accompanying items in the manual (CCMH, 2022). The Rasch analyses were performed on the joint data from 2001 to 2003 and 2021, so that the two hopelessness items were placed on a common metric anchored by the original MDI items. The new hopelessness item was assessed both dichotomized as a core item (for DSM‐5) and an accompanying item (for ICD‐11), and two dichotomizations were tested for the SCL hopelessness items. After subjecting the items to Rasch modeling the residuals were extracted and subjected to first a principal component analysis to examine unidimensionality, and second examined for correlation patterns (local dependence). Unidimensionality and local independence are prerequites for scaleability and essentially there should be little correlation patterns beyond that of the Rasch model. Eigenvalue for the first component in a PCA of the residuals is used to assess correlation beyond random variation, but this is sensitive to test length and sample size. The larger the samples and shorter the scales the critical value for strict unidimensionality approaches 1 (Linacre & Tennant, 2009). Correlation of residuals in item pairs is used to test for local independence similar to multidimensionality. A high positive correlation may indicate that items are too similarly formulated, that is, redundancy.

The Rasch analysis provides information on the severity of the individual items based upon latent trait modeling where person and item parameters are on the same scale. Equal probability of a positive response is modeled as a logistic function of the difference between the person and item parameter, and where person location (in logit) equals the severity of the item. The location on the latent trait, where there is a 0.5 probability of a positive response, is used to indicate severity of the item.

Rasch analysis typically also test the fit of the items to the model, by examining how the individual item observed logit curve slope differs from the overall modeled logit curve. One such test fit is the unstandardized mean squares (UMS), which has an expected value of one. Values above one indicates less good fit relative to the overall. A value below one indicates better than expected fit. A non‐parametric test of homogeneity of the items was also performed through Mokken analysis/modeling Loevinger coefficients for items and the scale (*H*_*i*_ and H). *H*_*i*_ assesses if an item is coherent enough to be included in the scale where a value larger than 0.3 is often used. *H* is often used as a measure of unidimensionality, where coefficients between 0.3 and 0.4 is considered a weak scale, 0.4–0.5 of medium strength and >0.5 a strong scale (Stochl et al., 2012).

Since two item pairs displayed local dependence the magnitude of the potential violation of independence was assessed by comparing a Rasch model where the items are assumed statistically independent to a model where the items hypothesized to be dependent were combined into polytomous items (summed). The two models were assessed for differences in reliability, person separation and item difficulty (Marais & Andrich, 2008). We expected that reliability and separation would decrease in the latter model if these were inflated, due to local dependence, in the former.

The difference in prevalence between DSM‐IV, DSM‐5, ICD‐10 and ICD‐11 depression according to MDI were examined separately for 2001–2003 and 2021. Agreement between the MDI classifications and clinical diagnosis (SCAN) was examined in the 2001–2003 sub‐sample through computations of sensitivity and specificity, and also Cohen's Kappa coefficients as measures of agreement. The coefficients range just like correlation coefficients between −1 (total disagreement) through 0 (at random) and 1 (total agreement). These were computed using weights to correct for the double‐phase sampling design. The description of the weights are provided elsewhere (Lundin, et al., 2015).

SAS, JMETRIK and the MOKKEN library in *R* were used for the statistical analyses.

## 3 RESULTS

Table 1 shows the results from the Rasch analysis of the ten dichotomized MDI items (ICD‐10) together with the two hopelessness items. Severity of the items ranged from −2.53 (sleep difficulties) to 1.82 (life not worth living). While severity refers to where on the latent scale (latent depression score) that the item is principally used, it is largely related to how common it is (higher severity = more uncommon). Fit of the items was good except for sleep difficulties and appetite. The MDI hopelessness item ranked just below the core symptoms depression and anhedonia and was the second most severe of the accompanying symptoms. The SCL hopelessness item was about half a logit less severe. While the SCL hopelessness item had acceptable fit, the new added MDI hopelessness item tended to overfit the model, possibly indicating high centrality or possibly strong correlation with other item or items. Since DSM‐5 hopelessness is part of the core symptom mood we also examined the severity of hopelessness dichotomized as a core item (Supplementary Table S2). This rendered the hopelessness ranking to the most (MDI) and third most severe items (SCL).

**TABLE 1:** Severity and fit of the ICD‐10 items and hopelessness.

Content	MDI item no	Severity	se	WMS	UMS
Teadium vitae	6	1.82	0.05	0.95	1.04
Depressed	1	0.96	0.04	0.93	0.82
Anhedonia	2	0.86	0.04	0.90	0.78
Hopelessness	New	0.65	0.05	0.91	0.70
Concentration	7	0.23	0.04	1.03	0.98
Guilt	5	−0.01	0.04	0.85	0.71
Energy	3	−0.02	0.04	0.88	0.76
Hopelessness	SCL	−0.08	0.05	1.05	1.09
Low confidence	4	−0.17	0.04	0.83	0.69
Appetite (low/high)	10	−0.64	0.03	1.41	1.67
Psychomotor	8	−1.07	0.03	0.89	0.85
Sleep problems	9	−2.53	0.03	1.24	1.70

*Note*: Severity is sometimes referred to as difficulty. The criteria are sorted by severity. New Hopelessness coded as an accompanying feature (0‐0‐1‐1‐1), SCL Hopelessness coded (0‐0‐0‐1‐1).

Abbreviations: se, Standard error; UMS, unweighted mean square; WMS, weighted mean square.

Examining the residuals for the ten dichotomized MDI items and the new hopelessness item in the 2021 data showed two item‐pairs that displayed strong correlation: item pairs 4 and 5 (feeling worthless and inappropriate guilt) and: item pairs 6 and 11 (life not worth living and hopelessness). The correlation matrix of the residuals is presented as Supplementary Table S3. Assessing the effect of this potential violation of local independence by comparing a Rasch model of all dichotomous items (assumed independent) to a model where these item pairs were analyzed as polytomous items showed little difference in reliability, separation and fit (Supplementary Table S4).

Coefficients from Mokken analysis are presented in Supplementary Table S5, showing that none of the items fell below the suggested threshold (*H*_*i*_) and that the summary coefficents (H) is to be regarded as a strong unidimensional scale (>0.5). Principal component analysis of the residuals from the Rasch analysis resulted in an eigenvalue for the first contrasting component of 2.15, indicating it explained more than random variation. This confirms potential multidimensionality, but in the strength of about 2 items and thus indicative of a dominant dimension with the possible presence of a smaller dimension. Hence, we believe that the MDI is not strictly unidimensional but essentially and sufficiently unidimensional.

Prevalence of depression according to the four classifications is shown in Table 2. There were no major differences between the four systems, but the change from MDI‐DSM‐IV to ‐DSM‐5 and from MDI‐ICD‐10 to ‐ICD‐11 rendered an increase in prevalence. The SCAN‐based classifications differed very little in prevalence across the systems. The MDI‐classifications agreement with SCAN ranged between Kappa 0.21–0.28, with high specificity and lower sensitivity.

**TABLE 2:** Prevalence of MDI and SCAN major depression and agreement with SCAN diagnoses.

	Prevalence				Agreement		
	MDI 2021 (*n* = 8863)	MDI 2000 (*n* = 8511)	MDI 2001–2003 Subset (*n* = 881)	SCAN 2001–2003 Subset (*n* = 881)	Sensitivity	Specificity	Kappa
	%	%	w%	w%	w%	w%	wK
DSM‐IV	6.7	5.1	4.7	1.8	55.0	96.2	0.29
DSM‐5	7.7	5.3	4.9	1.8	57.0	96.1	0.29
ICD‐10	6.5	5.2	4.8	2.4	58.6	96.5	0.37
ICD‐11	7.2	5.8	5.4	2.0	63.5	95.8	0.32

Abbreviations: MDI, Major Depression Inventory; SCAN, Schedules for Clinical Assessments in Neuropsychiatry.

In a secondary analysis we examined the effect of the changes only in the core criteria/features (Table 3). Adding hopelessness to sadness increased the caseness between DSM‐IV and DSM‐5. Lowering the cut off between ICD‐10 and ICD‐11 increased the caseness despite the removal of low energy.

**TABLE 3:** Prevalence of the core features according to the four systems.

	MDI 2021	MDI 2001–2003	MDI	SCAN
	(*n* = 8863)	(*n* = 8511)	2001–2003 sub.	2001–2003 sub.
	%	%	w%	w%
DSM‐IV^a^	9.4	8.4	7.0	6.4
DSM‐5^b^	11.2	9.2	7.4	6.7
ICD‐10^c^	6.9	5.7	5.0	2.9
ICD‐11^a^	9.4	8.4	7.0	6.4

Abbreviations: MDI, Major Depression Inventory; SCAN, Schedules for Clinical Assessments in Neuropsychiatry.

^a^ 1+ of Mood and Anhedonia.

^b^ 1+ of Mood/Hopeless and Anhedonia.

^c^ 2+ of Mood, Anhedonia or Energy.

The agreements between the four MDI classifications are presented in Table 4. In 2021, the highest agreement was between ICD‐11 and DSM‐IV, and the lowest between ICD‐10 and DSM‐5. In 2001–2003, ICD‐11 also had a strong agreement with DSM‐IV, while ICD‐10 generally had weaker associations with the three other systems.

**TABLE 4:** Kappa coefficient agreement between the MDI diagnostic systems in 2021 (upper) and 2000 (lower).

	Kappa coefficients			
	DSM‐IV	DSM‐5	ICD‐10	ICD‐11
DSM‐IV	‐‐‐	0.92	0.89	0.95
DSM‐5	0.97	‐‐‐	0.82	0.89
ICD‐10	0.89	0.87	‐‐‐	0.87
ICD‐11	0.93	0.91	0.88	‐‐‐

## 4 DISCUSSION

### 4.1 Main findings

In this study we constructed a hopelessness item for the MDI questions as a proposition to include such an item in the MDI to adhere to the operationalization of DSM‐5 and ICD‐11. Examining the MDI items, we found that all items except sleep problems and appetite had good fit. Hopelessness was a severe item, irrespective if scored as a core or accompanying criterion. The new hopelessness item had good fit to the model but displayed local dependence with not wanting to live. The prevalence and criterion validity of MDI DSM‐5 and ICD‐11 depression were assessed using a hopelessness question from SCL, which was more severe than the newly formulated hopelessness (i.e. more conservative). The changes in operationalization from DSM‐IV to DSM‐5 and ICD‐10 to 11 resulted in slightly increased prevalence and a slight decrease in sensitivity for ICD‐11.

### 4.2 Comparison with previous studies

Overall, the items in the MDI were found to have good psychometric properties, except items 9 (sleep) and 10 (appetite) that demonstrated misfit. This is in line with a similar Rasch analysis based on primary care patient in Denmark (Christensen et al., 2019) and finding based on Mokken analysis in depressed patients (Olsen, et al., 2003). The misfit of items 9 and 10 in our sample was also shown by Christensen and colleagues, in a primary care material, which were suggested to be due to non‐specificity of these items (overlap with somatic symptoms, anxiety, and medication). Homogeneity coefficients in our study agreed well with those reported previously (Bech et al., 2013; Olsen et al., 2003). The MDI algorithms in the present study showed sensitivities of 56%–67% and specificities above 95%. In comparison, Bech and colleagues found sensitivity/specificity of 90% and 82% (DSM–IV) and of 86% and 0.86% (ICD‐10) in a population of psychiatric patients matched with normal controls, all examined using SCAN (Bech, et al., 2001). A possible difference between our and their study might lie in the use of a general population sample. Screening scales applied to general population generally perform less well than in clinical samples (Eaton et al., 2007). A previous study based on PART the baseline MDI–DSM–IV algorithm was compared with SCAN‐DSM‐IV depression reported sensitivity similar to our findings (67%), but a specificity of only 22%. However, the contingency tables indicate that the specificity was about 81%. In an epidemiological setting such sensitivity and specificity produce a relatively large number of false positives, inflating prevalence and of course also diluting risk estimates.

### 4.3 Explaining the findings

In terms of severity the new hopelessness item ranked high, irrespective if dichotomized as a core or as an accompanying item. The SCL hopelessness had slightly lower severity and was similar to worthlessness and guilt. The higher severity of core symptoms is of course partly driven by the higher item cut off for dichotomization, but the similarity in severity between hopelessness, guilt and worthlessness is in line with findings by McGlinchey and colleagues (McGlinchey, et al., 2006), in a study where they examined DSM‐IV symptoms together with alternative symptoms of depression—a study that was cited when explaining the inclusion of hopelessness in ICD‐11 (Stein, et al., 2020). The dependence between hopelessness and not wanting to live is in concert with Beck's cognitive theory of depression where hopelessness and suicidal behavior are intimately connected (Beck et al., 1975). It is also in concert with Abramson's hopelessness theory of suicide (Liu et al., 2015). Item 4 (confidence) and 5 (guilt) displayed the strongest local dependence—and is also a compound criterion in DSM‐IV/5 and ICD‐11.

Including hopelessness in the sadness criteria, following the change in the symptom description in DSM‐5 was found to increase the number of persons fulfilling the core criteria of depression (at least using MDI), which resulted in an overall increase in proportion of depression. This partially justifies the criticism that this potentially broadens the diagnosis (Uher, et al., 2014). For ICD‐11 the changes are more dramatic. The removal of low energy from the core‐item set, together with a lowered threshold for the core features, does harmonize ICD‐11 depression with DSM, and increase the prevalence fulfilling the diagnosis. However, the increased cut off for accompanying symptoms renders no dramatic increase in prevalence (despite additional accompanying symptoms).

### 4.4 Implications

The MDI algorithms for DSM‐5 and ICD‐11 display similar criterion validity as DSM‐IV and ICD‐10. The operational changes between ICD‐10 and ICD‐11, and between DSM‐IV and DSM‐5 are on the face of it not dramatic, but while MDI‐DSM‐IV with confidence can be said to proxy DSM‐5, the changes in ICD‐11 brings this category closer to DSM‐IV than ICD‐10. Hence, the DSM‐IV is a better proxy for ICD‐11 than ICD‐10. However, updating the MDI item set to encompass hopelessness would be needed to ensure content validity. We believe that the MDI is a valuable tool for assessing depression and hope the developers would consider adding a hopelessness item to the item set. Since MDI could also be scored as a rating scale such a revision would have consequences beyond the diagnostic algorithms (increasing the range of scores). The MDI has previously undergone revisions to be able to measure atypical depression, why an additional item would not seem implausible (Bech, et al., 2013).

### 4.5 Methodological considerations

One weakness of the present study is that SCL hopelessness question was placed in a separate section of the questionnaire. Principally this is not a major issue since symptoms should ideally be independent and not have carry over effects. Hopelessness was included as part of SCL domains of depression, anxiety, and hostility, so it is likely that the interpretation was similar. However, the difference in response alternative was reflected in differences in severity. Part of the validation analysis hinges on that the SCL hopelessness item being an acceptable proxy for the MDI, which we believe it is when dichotomized. An additional weakness is that clinical assessments were not performed in 2021, when we added the new hopelessness item, but only in the older data waves. A direct assessment of criterion validity of the augmented MDI is thus still lacking. Awaiting for a psychiatric interview schedule for ICD‐11, future validation studies could use SCAN to operationalize a criterion standard, or alternatively that interviews which only cover DSM‐IV/5 and ICD‐10 criteria are completed with hopelessness.

Strengths of this study are that first, it is a large population‐based study with clinical symptoms assessed using semi‐structured interview that allowed for assessments of DSM‐5 and ICD‐11 operationalizations. Second, it is the first study attempting to update the MDI to cover DSM‐5 and ICD‐11 criteria. MDI is the only short “diagnostic” tool that covers both ICD and DSM criteria.

### 4.6 Conclusion

The new hopelessness item, formulated as “Have you felt that life is hopeless, your future appears bleak” had good psychometric properties, together with other MDI items. The new MDI algorithms for DSM‐5 and ICD‐11 increased the prevalence of depression but criterion validity remained stable. The operational change in ICD‐11 increased the agreement with DSM‐IV, making it more dissimilar to ICD‐10. There is hence a need for an MDI update to DSM‐5 and ICD‐11 standards and we conclude that such a revised version would provide good psychometric properties.

## CONFLICT OF INTEREST STATEMENT

None of the authors have any conflict to report.

## Supporting information

Supporting Information S1

## Data Availability

Research data are not shared.
