# Supplementary material for: The Major Depression Inventory for diagnosing according to DSM‐5 and ICD‐11: Psychometric properties and validity in a Swedish general population
Source: Int J Methods Psychiatr Res. 2023 Apr 12;32(4):e1966. doi: 10.1002/mpr.1966 (PMC10698816; doi:10.1002/mpr.1966)
Supplement: Supplementary file 1 — Supporting Information S1 [file MPR-32-e1966-s001.docx]

**Supporting information**

**Supplementary table S1.** SCAN items used to classify reference index of DSM-IV, DSM-5, ICD-10 and ICD-11.

|  | ICD-10 | ICD-11 | DSM-IV | DSM-5 |
| --- | --- | --- | --- | --- |
| Mood | 6.001, 6.003 | 6.001, 6.003 | 6.001, 6.003 | 6.001, 6.003, 6.006 |
| Anhedonia | 6.004 | 6.004 | 6.004 | 6.004 |
| Fatigue | 7.006, 7.007 | 7.006, 7.007 | 7.006, 7.007 | 7.006, 7.007 |
| Appetite/weight | 8.005, 8.006, 8.007 | 8.005, 8.006, 8.007 | 8.005, 8.006, 8.007 | 8.005, 8.006, 8.007 |
| Insomnia | 8.011, 8.013, 8.014, 8.016 | 8.011, 8.013, 8.014, 8.016 | 8.011, 8.013, 8.014, 8.016 | 8.011, 8.013, 8.014, 8.016 |
| Psychomotor | 7.005, 3.006 | 7.005, 3.006 | 7.005, 3.006 | 7.005, 3.006 |
| Worthless | 6.015, 6.017 | 6.015, 6.017  6.013 | 6.015, 6.017  6.013 | 6.015, 6.017  6.013 |
| Guilt | 6.013 |  |  |  |
| Concentr./  indecisive | 7.002, 7.003 | 7.002, 7.003 | 7.002, 7.003 | 7.002, 7.003 |
| Taedium vitae/  suicide | 6.010, 6.011, 6.012 | 6.010, 6.011, 6.012 | 6.010, 6.011, 6.012 | 6.010, 6.011, 6.012 |
| Hopeless |  | 6.006 |  | In mood |

Note: numbers refer to SCAN 2.1 item numbers.

**Supplementary table S2.** Severity and fit of the ICD-10 items and hopelessness.

| Content | Item no. | Severity | se | WMS | UMS |
| --- | --- | --- | --- | --- | --- |
| Hopelessness | SCL | 1.82 | 0.09 | 1.05 | 1.11 |
| Teadium vitae | 6 | 1.57 | 0.05 | 0.95 | 1.09 |
| Hopelessness | New | 1.28 | 0.06 | 0.96 | 0.71 |
| Depressed | 1 | 0.71 | 0.04 | 0.93 | 0.84 |
| Anhedonia | 2 | 0.61 | 0.04 | 0.89 | 0.77 |
| Concentration | 7 | -0.02 | 0.04 | 1.03 | 0.99 |
| Guilt | 5 | -0.26 | 0.04 | 0.86 | 0.73 |
| Energy | 3 | -0.27 | 0.04 | 0.87 | 0.75 |
| Low confidence | 4 | -0.42 | 0.04 | 0.83 | 0.70 |
| Appetite (low/high) | 10 | -0.89 | 0.03 | 1.4 | 1.68 |
| Psychomotor | 8 | -1.33 | 0.03 | 0.88 | 0.85 |
| Sleep problems | 9 | -2.80 | 0.03 | 1.23 | 1.70 |

Note: Severity is sometimes referred to as difficulty, se = Standard error. WMS = weighted mean square UMS = unweighted mean square. The criteria are sorted by severity. New Hopelessness coded as a core feature (0-0-0-1-1), SCL Hopelessness coded (0-0-0-0-1)

**Supplementary table S3.** Correlation between residuals from the Rasch analysis of 11 MDI items .

| MDI Item (no.) | (1) | (2) | (3) | (4) | (5) | (6) | (7) | (8) | (9) | (10) | Hope-less |
| --- | --- | --- | --- | --- | --- | --- | --- | --- | --- | --- | --- |
| Depressed (1) | 1 |  |  |  |  |  |  |  |  |  |  |
| Anhedonia (2) | 0.02 | 1 |  |  |  |  |  |  |  |  |  |
| Energy (3) | -0.02 | 0.19 | 1 |  |  |  |  |  |  |  |  |
| Low confidence (4) | -0.13 | -0.09 | -0.13 | 1 |  |  |  |  |  |  |  |
| Guilt (5) | -0.10 | -0.14 | -0.21 | **0.37** | 1 |  |  |  |  |  |  |
| Teadium vitae (6) | -0.03 | -0.12 | -0.18 | -0.07 | -0.03 | 1 |  |  |  |  |  |
| Concentration (7) | -0.17 | -0.13 | -0.16 | -0.15 | -0.10 | -0.03 | 1 |  |  |  |  |
| Psychomotor (8) | -0.12 | -0.09 | -0.03 | -0.15 | -0.17 | -0.12 | 0.04 | 1 |  |  |  |
| Sleep problems (9) | -0.06 | -0.10 | -0.06 | -0.22 | -0.21 | -0.13 | -0.12 | -0.14 | 1 |  |  |
| Appetite (10) | -0.13 | -0.15 | -0.14 | -0.18 | -0.20 | -0.13 | -0.08 | -0.14 | -0.07 | 1 |  |
| Hopeless | -0.04 | -0.19 | -0.20 | -0.09 | -0.03 | **0.23** | -0.09 | -0.13 | -0.12 | -0.11 | 1 |

Note: Variables 4 and 5 strongly correlated (Low confidence and Guilt) but also Hopeless and item 6 (Hopeless and Teadium vitae).

**Supplementary table S4** Severity and fit of the ICD-10 and hopelessness items, with and without super-items.

|  |  | Only dichotomous items  (model 1) | | | |  | With higher-order polytomous items (model 2) | | | |
| --- | --- | --- | --- | --- | --- | --- | --- | --- | --- | --- |
| Content | Item no. | Severity | se | WMS | UMS |  | Severity | se | WMS | UMS |
| Depressed | 1 | 1.32 | 0.06 | 0.95 | 0.92 |  | 1.40 | 0.06 | 0.91 | 0.82 |
| Anhedonia | 2 | 0.99 | 0.06 | 0.90 | 0.73 |  | 1.08 | 0.06 | 0.86 | 0.68 |
| Energy | 3 | -0.01 | 0.05 | 0.93 | 0.85 |  | 0.14 | 0.05 | 0.89 | 0.80 |
| Low confidence | 4 | -0.32 | 0.05 | 0.85 | 0.74 |  |  |  |  |  |
| Guilt | 5 | -0.09 | 0.05 | 0.83 | 0.70 |  |  |  |  |  |
| Low confidence/Guilt | HO poly1† |  |  |  |  |  | -0.22 | 0.03 | 0.95 | 0.83 |
| Teadium vitae | 6 | 1.61 | 0.07 | 0.94 | 1.04 |  |  |  |  |  |
| Hopelessness | New | 0.64 | 0.05 | 0.91 | 0.70 |  |  |  |  |  |
| Teadium vitae/ Hopelessness | HO poly 2‡ |  |  |  |  |  | 0.99 | 0.04 | 0.97 | 0.85 |
| Concentration | 7 | 0.02 | 0.05 | 1.0 | 0.95 |  | 0.16 | 0.05 | 0.95 | 0.88 |
| Psychomotor | 8 | -1.14 | 0.04 | 0.90 | 0.88 |  | -0.94 | 0.04 | 0.87 | 0.83 |
| Sleep problems | 9 | -2.37 | 0.04 | 1.29 | 1.79 |  | -2.13 | 0.04 | 1.25 | 1.56 |
| Appetite (low/high) | 10 | -0.65 | 0.05 | 1.37 | 1.63 |  | -0.47 | 0.04 | 1.29 | 1.44 |
|  |  |  |  |  |  |  |  |  |  |  |

Note: comparisons made in the 2021 sample. †=Higher order polytomous, combining (sum of) Low confidence and Guilt, i.e. score 0-2. ‡ = Higher order polytomous, combining (sum of) Teadium vitae and Hopelessness, i.e. score 0-2. Person separation index: model 1 = 1.34, model 2 = 1.20. Person Reliability: model 1 = 0.64, model 2 = 0.59. Item separation index: model 1 = 21.1, model 2 = 22.5. Item reliability: model 1 = 0.998, model 2 = 0.998. The Person separation index and Person Reliability indicate that measure is only sensitive enough to distinguish between 1-2 groups of severity, which is due to a relatively limited number of items and a homogenous sample (many with similar, low, values on the latent trait). The high Item separation and item reliability indicate that the sample is large enough to precisely locate the items on the latent trait.

**Supplementary table S5.** Mokken analysis of MDI ICD-11 items in 2021.

| Content | MDI Item no. | H (se) | 95% CI |
| --- | --- | --- | --- |
| Depressed | 1 | 0.62 (0.013) | (0.59–0.64) |
| Anhedonia | 2 | 0.63 (0.011) | (0.61–0.65) |
| Energy | 3 | 0.60 (0.010) | (0.58–0.62) |
| Low confidence/Guilt | 4 or 5 | 0.62 (0.010) | (0.60–0.64) |
| Teadium vitae | 6 | 0.64 (0.015) | (0.61–0.67) |
| Concentration | 7 | 0.58 (0.010) | (0.56–0.60) |
| Psychomotor | 8 | 0.66 (0.010) | (0.64–0.68) |
| Sleep problems | 9 | 0.66 (0.012) | (0.64–0.68) |
| Appetite (low/high) | 10 | 0.49 (0.012) | (0.47–0.51) |
| Hopeless | New | 0.61 (0.011) | (0.59–0.63) |
|  |  |  |  |
|  |  | Scale H (se) | 95% CI |
|  |  | 0.61 (0.008) | (0.59–0.62) |
